# An Integrative Systematic Approach Reveals a New Species of *Crocus* Series *Verni* (Iridaceae) Endemic to Albania

**DOI:** 10.3390/plants14050741

**Published:** 2025-02-28

**Authors:** Irena Raca, Donald Shuka, Lulëzim Shuka, Nomar Espinosa Waminal, Dörte Harpke

**Affiliations:** 1 Department of Biology and Ecology, Faculty of Sciences and Mathematics, University of Niš, 18000 Niš, Serbia; 2 Department of Biology, Faculty of Technical Sciences, University of Vlora “Ismail Qemali”, 9401 Vlorë, Albania; donald.shuka@univlora.edu.al; 3 Department of Biology, Faculty of Natural Sciences, University of Tirana, Bld “Zogu I” Nr. 25/1, 1001 Tiranë, Albania; lulezim.shuka@fshn.edu.al; 4 Leibniz Institute of Plant Genetics and Crop Plant Research (IPK), 06466 Gatersleben, Germany; waminal@ipk-gatersleben.de (N.E.W.); harpke@ipk-gatersleben.de (D.H.)

**Keywords:** *Crocus* series *Verni*, new species, phylogeny, genotyping-by-sequencing (GBS), fluorescence in situ hybridization (FISH), chromosome numbers, morphology, leaf anatomy

## Abstract

The allopolyploid complexes in *Crocus* series *Verni* represent taxonomic challenges due to their variable or mostly overlapping morphology with one parental species. Moreover, their diploid ancestors remain unidentified, even with genome-wide SNP data. One such case, collected from the southeasternmost point of the series’ geographical distribution, is herein characterised and described as a new species, *C. bachofenii*. This study integrates phylogenomics and cytogenetics to infer the parental origin of *C. bachofenii* and establish its diagnostic morphological characteristics. Genome skimming of *C. bachofenii* and 10 other *C.* ser. *Verni* species enabled the development of novel satellite repeats as cytogenetic markers and the assembly of their complete chloroplast genomes that were employed for phylogenetic analysis alongside GBS data. The allopolyploid origin of *C. bachofenii* (2*n* = 16) was confirmed with *C. vernus* as the maternal parent. The probably extinct paternal parent was affiliated with a clade comprising *C. heuffelianus*, *C. tommasinianus*, *C. kosaninii*, and *C. bertiscensis*. Morphologically, *C. bachofenii* is distinguished by larger flowers, perigone segment coloration, and a stigma–anther ratio from its close relatives. In conclusion, its phylogenetic affiliation, distinctive cytological status, and unique morphological features justified the description of this taxon as a new species.

## 1. Introduction

Allopolyploid complexes are taxonomically challenging for various reasons [1]. Their high morphological variability can lead to characters overlapping with other species [2], or a predominance of one of the parental genomes can result in a high resemblance to one of the parents. Moreover, it might complicate the morphological recognition of the allopolyploids [3,4].

In the past decade, *Crocus* L. series *Verni* B. Mathew was the object of different investigations aimed at resolving phylogenetic relationships and unraveling the evolutionary history of allopolyploids and their cytotypes [4,5]. The group currently comprises 12 species from Central and South Europe (*C. bertiscensis* Raca, Harpke, Shuka and V. Randjel., *C. etruscus* Parl., *C. ilvensis* Peruzzi and Carta, *C. heuffelianus* Herb., *C. kosaninii* Pulević, *C. longiflorus* Raf., *C. neapolitanus* (Ker Gawl.) Loisel., *C. neglectus* Peruzzi and Carta, *C. siculus* Tineo, *C. tommasinianus* Herb., and *C. vernus* (L.) Hill) and the allotetraploid complex of *C. heuffelianus* and *C. vernus* hybrids.

While disentangling the latter allopolyploid complex [4], a tetraploid “*C*. cf. *vernus*” 2*n* = 16 was found in Central and Northeastern Albania. Analysis of three chloroplast markers indicated that *C. vernus* 2*n* = 8 was the maternal parent [4]. However, it remained unclear whether this tetraploid emerged as an autoploid or alloploid. To address these issues, we added an additional analysis of genome-wide single nucleotide polymorphism (SNP) data obtained by genotyping-by-sequencing (GBS; [6]) and skimmed the genomes of *C. bachofenii* and nine other *C*. ser. *Verni* species for novel satellite repeats that can be used as cytogenetic markers. Such cytogenetic markers can provide insights into the polyploidy origin, i.e., autopolyploidy versus allopolyploidy, by revealing the number of chromosomes with similar chromosomal distribution patterns as a function of polysomic versus disomic inheritance, respectively [7,8]. Moreover, these cytogenetic markers could enlighten us about the complex karyotype evolution in *C*. ser. *Verni*, which is characterised by an extensive degree of chromosomal rearrangements resulting in different chromosome numbers. Therefore, we also characterise the karyotype. Genome skimming data were also used to compare the entire chloroplast of most *C*. ser. *Verni* species aimed to arrive at a higher resolution in comparison to [4].

Additionally, detailed morphological investigations were also lacking [4], which would have allowed a correct taxonomical treatment. Therefore, thorough morphological investigation and field studies were conducted to establish diagnostic characteristics essential for clear identification and circumscription of the distribution. Our morpho-anatomical investigation initially included the known species of *C*. ser. *Verni* within the geographical area surrounding the new species. So far in Albania, only *C. bertiscensis* and *C. vernus,* as well as the allotetraploid *C.* cf. *heuffelianus,* have been reported for *C*. series *Verni*, with their distribution in the alpine region of the Albanian Alps at altitudes ranging from 850 m to 2300 m a.s.l. [9,10]. Moreover, Meyer [11] also reported the occurrence of *C. tommasinianus* in Kunora e Lurës.

## 2. Results

### 2.1. Phylogenetic Affiliation

**Figure 1:**
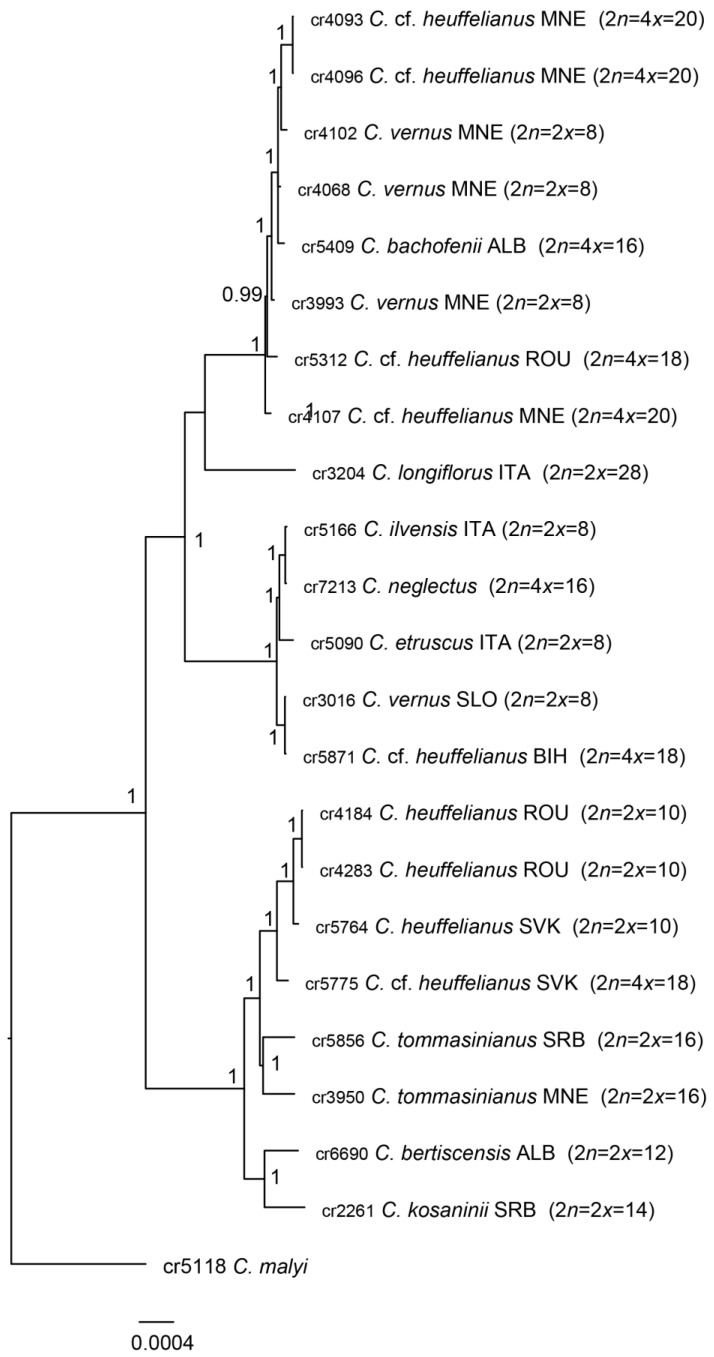
The phylogenetic tree was constructed using Bayesian inference implemented in MrBayes 3.2.7 based on whole chloroplast genome data. Posterior probabilities, representing the statistical confidence of each node, are indicated at each branching point. Each species is labeled with the DNA extraction ID preceding the species name, followed by the country ISO code for the sample origin, ploidy level, and chromosome number.

**Figure 2:**
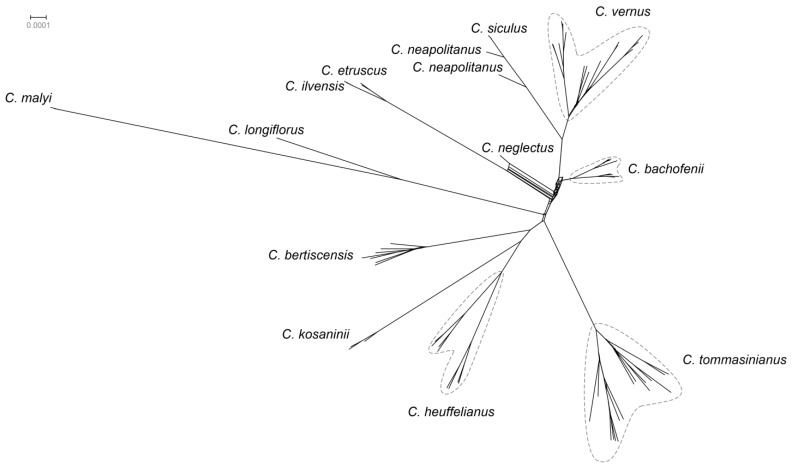
SuperNetwork generated in SplitsTree 4.18.2 shows gene tree incongruence based on 500 maximum likelihood trees estimated by IQ-tree v2.2.6 using SNP data (74 sequences, 19,435 SNPs, 10,831 parsimony informative sites).

*Crocus bachofenii* shares the nearly identical chloroplast haplotype with the eastern *C. vernus* (Figure 1). A supernetwork was constructed using SplitsTree based on 500 rooted maximum likelihood trees obtained by IQ-tree. It depicts *C. bachofenii* positioned near the centre of the network, with *C. vernus* being the closest diploid species, followed by *C. heuffelianus* (based on the mean branch lengths of the 500 trees) (Figure 2).

*Crocus bachofenii* possesses 1674 private alleles out of 32,170 in the dataset (Table S1), which otherwise includes only diploid species from *C.* ser. *Verni*. Consequently, 30,378 alleles of *C. bachofenii* are shared with other *C*. ser. *Verni* species, of which 99.6% are found in either the cladeI (*C. vernus*, *C. siculus,* and *C. neapolitanus*) or cladeII (*C. bertiscensis*, *C. heuffelianus*, *C. kosaninii*, and *C. tommasinianus*). *Crocus bachofenii* shares 87% of its none-private SNPs with both cladeI and cladeII, respectively. Specifically, it shares 85% (25,851 SNPs) of its none-private SNPs with *C. vernus*, among which 1777 SNPs are exclusively shared between these two species (Table 1). *Crocus heuffelianus* and *C. tommasinianus* share 69% (21,187 SNPs) and 60% (18412 SNPs), respectively, with *C. bachofenii*, including 550 SNPs (with *C. heuffelianus*) and 289 SNPs (with *C. tommasinianus*) that are exclusively shared. All other diploid species share less than 50% of their SNPs with *C. bachofenii* (Table 1).

**Figure 3:**
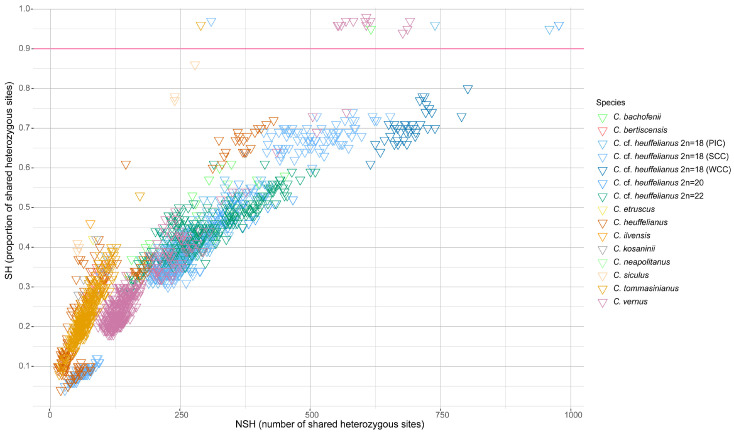
Heterozygosity point plot based on all intraspecific sample pairs. Each data point represents one pair of samples. The sample pairs of each species and the different *C.* cf. *heuffelianus* allotetraploids, respectively, are indicated by different colours. For tetraploids *C.* cf. *heuffelianus* with 2*n* = 4*x* = 18, the geographic affiliation is also provided, as Pannonian–Illyric Clade (PIC), Southern Carpathian Clade (SCC), and Western Carpathian Clade (WCC). The pink line indicates the threshold of 90% to clone pairs from none-clone pairs.

The shared heterozygosity was used to infer the degree of clonal reproduction in *C. bachofenii*. Only one clonemate pair was found within the Mt. Zeba population of *C. bachofenii*; otherwise, shared heterozygosity was 0.61 or lower (Figure 3).

### 2.2. Karyological Analysis

**Figure 4:**
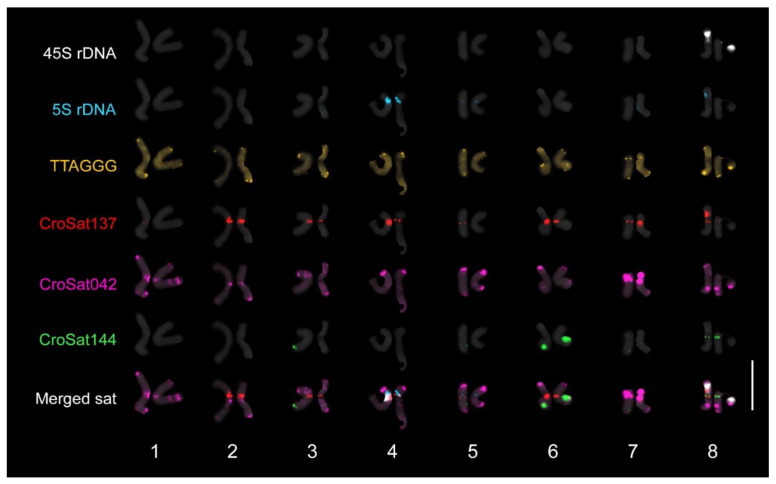
FISH karyogram of *C. bachofenii* using six cytogenetic markers. Note the hemizygous distribution of 5S rDNA at the long arm of chromosome 3, CroSat137 in chromosomes 1 and 5, and CroSat144 in chromosomes 3, 5, and 7. Bar = 10 µm.

*Crocus bachofenii* has a chromosome number of 2*n* = 16 (Figure 4) and genome size of 2C = 12.25 ± 0.19 pg (*Pisum sativum* L. was used as standard; CV standard: 2.73–3.72; CV sample: 4.47–7.38) (taken from Table S3 published by [4]).

We identified three high-confidence satellite repeats named CroSat137, CroSat042, and CroSat144, each with consensus sequence lengths of 159, 178, and 186 bp (Figure S1), respectively, that were used in addition to the 5S rDNA, 45S rDNA, and TTAGGG telomeric repeat for FISH [12].

The six FISH probes showed a range of heterozygous distribution (Figure 4 and Figure S2). The 45S rDNA was localised in only one chromosome pair at the short arm of chromosome 8. Five signals were observed for the 5S rDNA, including one major pair at the short arm of chromosome 4, one minor pair at the short arm of chromosome 5, and a hemizygous locus at the long arm of chromosome 3. The TTAGGG telomeric repeat, while showing distinct signals at the ends of the chromosomes, showed a dispersed distribution along all chromosomes. The CroSat137 showed a putative centromeric distribution in 14 out of the 16 chromosomes. Six pairs (chrs. 2–4, 6–8) have CroSat137 signals in both chromosomes, whereas chrs. 1 and 5 have hemizygous signals. Moreover, signal intensities also varied within a pair. For example, chrs. 4 and 7 have one homolog with a significantly more intense CroSat137 signal than another homolog. The CroSat042 was detected in all chromosomes at subtelomeric, interstitial, and pericentromeric regions. The CroSat144 was detected in eight chromosomes, two homozygous loci (chrs. 6 and 8), and four hemizygous loci (chrs. 3, 5, and 7).

### 2.3. Taxonomic Treatment—Crocus bachofenii D. Shuka, Raca, and Harpke sp. nov.

#### 2.3.1. Type

Type: —ALBANIA, Central Albania, Tirana district, Linos meadows and Mali me Gropa (ca. 25 km far from Tirana)., i It was found on *Festuco-Brometea* subalpine pastures and meadows on limestone substrate, 1300–1700 m a.s.l. on 9 May 2019, *D. Shuka* (holotype GAT-83183! isotypes, TIR-09749! and Shuka *herb*.—003245 (GAT—Herbarium of Leibniz Institute of Plant Genetics and Crop Plant Research (IPK), Gatersleben, Germany; TIR—National Herbarium of Tirana, Albania; Shuka—Herbarium of Lulëzim and Donald Shuka, Tirana, Albania)).

#### 2.3.2. Description

**Figure 5:**
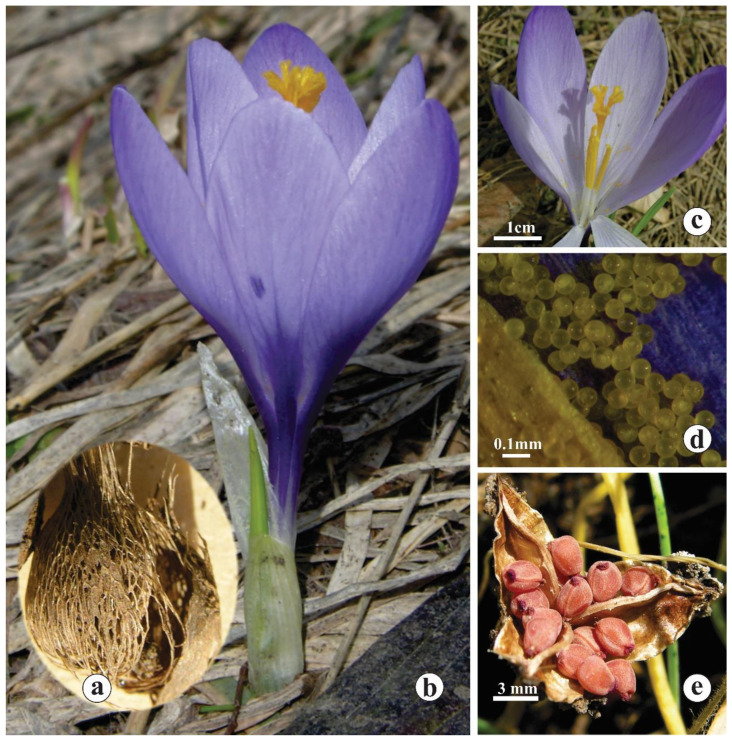
The morphology of *C. bachofenii* generative organs: (**a**) corm details; (**b**) the flower and habitus; (**c**) the hairy throat and ratio between anthers and stigma; (**d**) pollen grains; (**e**) the capsule with seeds.

Early spring species with subglobose to globose corms, 7–13 (10.60 ± 2.77) mm in diameter, covered with three-to-six layers of reticulated and parallel fibres 0.09–0.11 (0.10 ± 0.04) mm thick. Some fibres in the first third of the upper part of the corm are thicker than those appearing in its lower part (Figure 5a). The outer layers of tunics consist of free reticulated fibres, while the inner ones are composed of parallel and/or reticulated fibres that are connected by two different membranes, which are semi-transparent and thicker (Figure S3a,b,c). The neck is indistinct.

Cataphylls (sheathing leaves) were 3–5 (3.60 ± 0.00), white togreenish, with green veins at the apex (Figure 5b). Prophyll was present. The bract was membranous, whitish and transparent, often extended above the perigone throat. Bracteole was absent.

The perigone tube was white-to-whitish underground and violet-to-deep violet aboveground, while the throat was pale lilac and hairy (Figure 5c). The flowers were solitary, rarely in pairs, with perigone segments 30–50 (38.50 ± 1.00) mm long and 10–20 (14.50 ± 1.50) mm wide (the perigone segment dimensions were derived from both outer and inner segments). The perigone segments were oblanceolate, and rarely subobtuse to subacute with faint violet veins, which were more prominent in the inner segments (Figure 5b,c). The outer segments were equal to or slightly longer than the inner ones (Figure 5b,c); the ¾ length of the outer segments overlaps with the inner ones (Figure 5b). The outer segments were lilac-to-violet, rarely pale lilac or deep violet, with the coloration paler compared to one occurring in the inner ones (Figure 5b,c). The basal markings (represented by the intensive coloration) were present, while the apical ones were lacking (Figure 5b).

Filaments were whitish, 10–15 (11.54 ± 1.90) mm long (Figure 5c), and glabrous. Anthers were yellow, 13–18 (15.67 ± 3.00) mm long (Figure 5c) with spherical pollen grains, and 0.07–0.82 (0.08 ± 0.01) mm in diameter (Figure 5d).

Styles were orange in the upper parts and divided into three lobes 25–33 (28.84 ± 4.50) mm long (Figure 5c); stigma sublobes with a length of 0.14–0.35 (0.24 ± 0.01) mm can be noticed as well. The stigma is usually longer for 1.47 ± 2.5 mm (90% of cases), or rarely equal when compared to anthers (Figure 5c).

Capsules were 10–15 (13.40 ± 3.00) mm long, while the seeds were brown and globose-to-obovate, 2.84 × 2.63 mm (Figure 5e).

**Figure 6:**
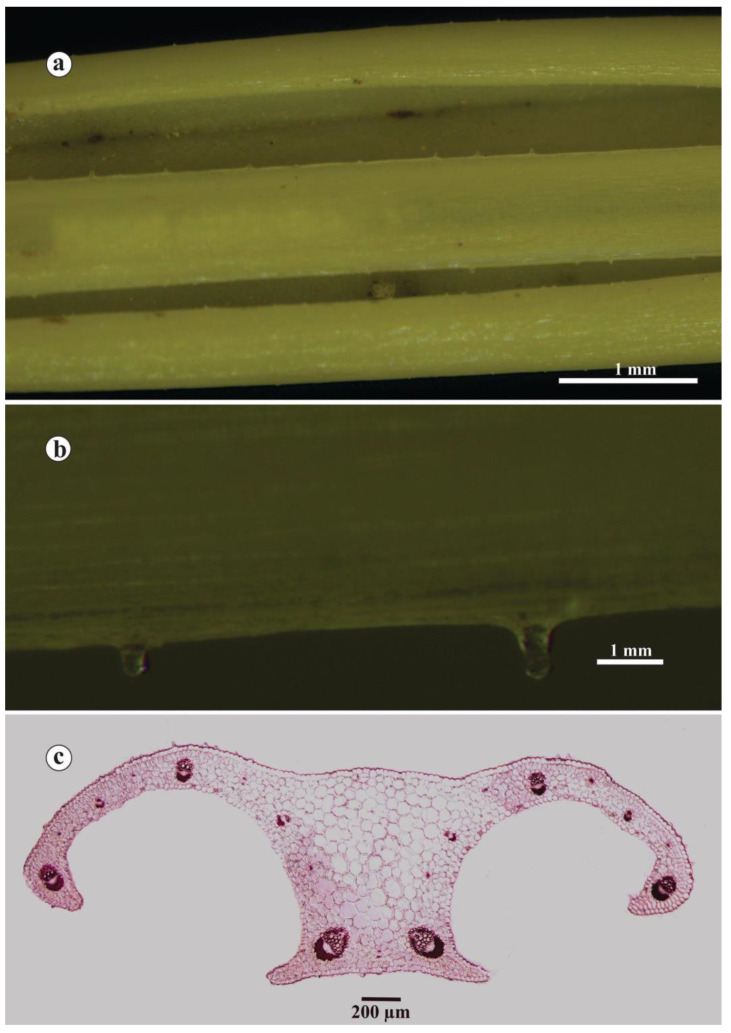
Leaves of *C. bachofenii*: (**a**) abaxial side appearance; (**b**) abaxial papillae detail; (**c**) transverse cross-section.

The leaves were two-to-five (2.84 ± 0.50) in number and 1.50–7.00 (2.93 ± 0.30) mm wide, mostly shorter than flowers. They were rarely longer, green with papillae at the basal corners of the leaves, and each one was composed of one or two cells 0.033–0.07 mm long (Figure 6a,b). The details of the transverse cross-section are illustrated in Figure 6c. Of the total leaf diameter—4740.88 ± 835.32 μm, 1980.60 ± 338.66 μm represents the average arm length, and 792.87 ± 222.83 μm is the white stripe width. The ratio of the leaf diameter compared to the white stripe width is 6.19 ± 1.10, the height of the keel is 776.63 ± 128.69 μm, and the central parenchyma area is 285560.70 ± 114763.65 μm^2^. The adaxial epidermal layer is about 23.58 ± 4.52 μm thick, with its cells about 20.05 ± 3.14 μm wide. On the other hand, the value of 23.02 ± 3.10 μm represents the height of the abaxial epidermal layer, while the value of 25.22 ± 4.12 μm represents the abaxial cell width. The palisade tissue of the leaf mesophyll (62.73 ± 10.19 μm) is thinner compared to the spongy layer (72.80 ± 23.05 μm). The palisade cell height is 38.43 ± 4.20 μm, and the width is 19.16 ± 1.93 μm. Furthermore, the value of 22.31 ± 4.02 μm refers to the height and 29.33 ± 3.81 μm to the width of the cells of the mesophyll spongy layer. The number of vascular bundles ranges from 17 to 25. The xylem area (1603.18 ± 426.24 μm^2^) is more prominent when compared to the phloem one (1234.55 ± 289.74 μm^2^). Sclerenchyma caps are well developed (3118.72 ± 1289.11 μm^2^).

The closest relative of *C. bachofenii* is *C. vernus* from the eastern distribution range according to the chloroplast phylogeny, while the GBS data suggest a close affiliation to the three species from the *C. vernus* complex (*C. vernus*, *C. neapolitanus,* and *C. siculus*). Morphologically, it resamples more *C. neapolitanus,* as well as *C. bertiscensis* and *C. tommasinianus* (Figures S3a–e and S4a–e), which supports the progeny from an extinct species of the *C. heuffelianus*, *C. tommasinianus*, *C. bertiscensis*, and *C. kosaninii* clade, in addition to the *C. vernus* complex. Compared to *C. vernus* (Figures S3e and S4c), *C. bachofenii* can be distinguished by bigger flowers, the different coloration of perigone segments, and a stigma longer than anthers or rarely equal to (Table 2, Figure S4c). The new species shares affinities in several morphological characters with *C. bertiscensis*, excluding the presence of a dark violet heart (or V)-shaped apical mark and short perigone tubes (Table 2 and Figure S4d). *Crocus bachofenii* differs from *C. tommasinianus* by the absence of the layers with parallel and thinner fibres of the corm tunics, the white or whitish perigone tubes, smaller perigone segments, and the absence of starry-shaped perigones and longer stigma sublobes (Table 2, Figures S3d and S4e). *Crocus neapolitanus* can be distinguished from the new species by having tunics with thicker fibers, shorter bract, and stigma sublobes, as well as shorter perigone segments lacking faint violet veins (Table 2, Figure S4b). The Italian allotetraploid *C. neglectus* (probably *C. neapolitanus* × *C. ilvensis* [5]) shares the same chromosome number as *C. bachofenii,* which is also similar to *C. bachofenii* but can be distinguished by characters that *C. neglectus* and *C. neapolitanus* shared with each other. *Crocus neapolitanus* and *C. neglectus* have bigger corms (11.9 mm and 13.9 mm, respectively), wider leaves and fewer cataphylls (2–3), longer perigone tubes, and shorter anthers (11.8 mm and 12.6 mm, respectively) and stigma sublobes (0.06 mm and 0.13 mm, respectively) than *C. bachofenii*.

#### 2.3.3. Etymology

The new species is named after Professor Reinhard Bachofen, from the Department of Plant and Microbial Biology at the University of Zürich, Switzerland, since the first record of the species refers to field trip investigations of L. Shuka and R. Bachofen in Kunora e Lurës (Lura-Mali i Dejës NP) on 15 April 2013.

#### 2.3.4. Phenology

The flowering time is March–May, depending on the altitude and exposition.

#### 2.3.5. Ecology and Distribution

*Crocus bachofenii* was only found in Albania and represents the southernmost distribution range of *C*. ser. *Verni* that could be confirmed up to now (Figure S5 and Table S2). It is growing in the subalpine calcareous rocky grasslands and meadows, or serpentinous pastures, of Central and Northeastern Albania (Figure S5). The new species prefers xerophilous and mesophilous grassland communities of the *Festuco-Brometea* class that occur in the alluvial depressions and meadows found in between openings of the beech belt at altitudes of 1300–1700 m a.s.l., which are characterized by a deep layer of soil. The grasslands and meadows of Mali me Gropa, Linos, Livadhet e Ketit, and Qafa e Qershisë (Dajti Mt), according to [13], are represented by *Trisetetum flavescens-Plantago media* and *Anthoxantho-Agrostietum* plant communities. In these meadows, *C. bachofenii* was accompanied by *Agrostis capillaris* L., *Anthoxanthum odoratum* L., *Barbarea balcana* Pančić, *Bellis perennis* L., *Brachypodium sylvaticum* (Huds.) P.Beauv., *Briza media* L., *Carex echinata* Murray, *Carex* sp. pl., *Colchicum autumnale* L., *Crepis biennis* L., *Cynosurus cristatus* L., *Dactylis glomerata* L., *Dactylorhiza cordigera* (Fr.) Soó, *Galanthus reginae-olgae* Orph., *Galium verum* L., *Geranium sylvaticum* L., *Filipendula vulgaris* Moench, *Muscari botryoides* (L.) Mill., *Poa pratensis* L., *Plantago media* L., *Ranunculus polyanthemos* L., *Rumex* sp., *Poterium sanguisorba* L., *Taraxacum* F.H. Wigg. sect. *Taraxacum*, *Trifolium pratense* L., *Trifolium repens* L., *Trisetum flavescens* (L.) P.Beauv., *Veratrum nigrum* L., *Veronica chamaedrys* L., and *Viola aetolica* Boiss. and Heldr. The xerophytic vegetation of the *Festuco-Brometalia* class in the Bjeshka e Oroshit, Mali me Gropa, and Zeba Mt localities belongs to the *Brometum erecti* and *Festucetum valesiacae* plant communities. The indicator plants in this habitat type are *Aristolochia pallida* Willd., *Asphodelus albus* Mill., *B. pinnatum* (L.) P. Beauv., *Bromopsis erectas* (Huds.) Fourr., *Euphorbia cyparissias* L., *Festuca valesiaca* Schleich. ex Gaudin, *Gagea* sp., *Gymnadenia conopsea* (L.) R. Br., *Helleborus odorus* Waldst. and Kit., *Koeleria splendens* C.Presl, *Leucanthemum vulgare* Lam., *Lilium albanicum* Griseb., *Narcissus poeticus* L., *Orchis mascula* (L.) L., *O. quadripunctata* Cirillo ex Ten., *Ornithogalum* sp., *Pedicularis brachyodonta* Schloss. and Vuk., *Poa bulbosa* L., *Plantago lanceolata* L., *Potentilla* sp., *Primula veris* L., *Thymus striatus* Vahl., and *Viola schariensis* Erben etc. Three localities of *C. bachofenii* in Mali me Gropa, Mali i Murrizës, and Qafa e Qershisë (Dajti Mt) are located within the Bovilla watershed and are strongly indicated by a Mediterranean climate [14]. The only locality reported from ultramafic substrates known so far occurs in Lura-Mali i Dejës National Park.

## 3. Materials and Methods

### 3.1. Plant Material

Plant material was initially collected in the framework of our previous study [4] (Table S3). Additional material from Albania was included as well (Table S2). The collection trips were conducted during the flowering time in 2022, 2023, and 2024.

### 3.2. DNA Extraction

Total genomic DNA was extracted from silica gel-dried leaf tissue with the DNeasy Plant Mini Kit (Qiagen) according to the instructions of the manufacturer. After DNA extraction, the DNA quality and concentration were checked on 1% agarose gels.

### 3.3. Library Preparation and Sequencing

Genomes of the new species *C. bachofenii* (one individual), all other *C*. ser. *Verni* species (except for *C. neapolitanus* and *C. siculus*), and the sister species to *C*. ser. *Verni C. malyi* were sequenced at a targeted coverage of 1–5×. Thus, one individual each of *C. bertiscensis*, *C. etrucus*, *C. ilvensis*, *C.* cf. *heuffelianus* 2*n* = 18 BIH, *C.* cf. *heuffelianus* 2*n* = 18 ROU, and *C. neglectus*; two individuals of *C. vernus*; three individuals of *C. heuffelianus*; one individual of *C.* cf. *heuffelianus* 2*n* = 18 SVK; and three individuals of *C.* cf. *heuffelianus* (2*n* = 20 MNE) were sequenced (Table S3). They were either processed by BGI (BGI Tech Solutions Co., Ltd., Shenzhen, China) and sequenced in a DNBseq system (MGI Tech Co., Ltd., Shenzhen, China) using a short-insert library in a 2 × 150 cycle or sequenced in-house. For the latter, library preparation was conducted as described by [15]). One-to-two µg of DNA were fragmented to 400–600 bp using Covaris S220 Focused-ultrasonicator (Covaris, MA, USA). Fragment size distribution and DNA concentration were evaluated on an Agilent BioAnalyzer High-Sensitivity DNA Chip (Agilent Technologies, Santa Clara, CA, USA) using the Qubit DNA Assay Kit in a Qubit 2.0 Fluorometer (Thermo Fisher Scientific, Life Technologies brand, Waltham, MA, USA). Finally, the DNA concentration of the libraries was checked by a quantitative PCR run. Cluster generation on Illumina cBot and paired-end sequencing (2 × 250 bp) on the Illumina NovaSeq6000 platform (SP reagent kit, v1.5 chemistry) followed Illumina’s recommendation and included 1% Illumina PhiX library as internal control (Illumina, San Diego, CA, USA).

For GBS, library preparation was conducted for a newly collected population of *C. tommasinianus* from Albania, along with three samples of *C. siculus*, following the protocol outlined in [4].

Barcoded reads were de-multiplexed using the Casava pipeline 1.8 (Illumina). The obtained raw sequencing reads were quality-checked and overrepresented, i.e., clonal reads were detected with FastQC v.0.12.1 [16]. Adapter trimming of sequence reads was performed with Cutadapt v.3.3 [17], and reads shorter than 120 bp after adapter removal for WGS were discarded, or, within the IPYRAD v.0.9.58 [18] pipeline, reads shorter than 80 bp were discarded after adapter removal.

### 3.4. Assembly of Chloroplast Genomes

GetOrganelle v1.7.7.0 [19] was used to de novo assemble a first draft of the plastid genome. This toolkit implements Bowtie 2 [20] to initially find reads mapping to a plant chloroplast database and SPAdes [21] for de novo assembly and iterative extension. During the assembly and iteration process, Blast+ [22] is used to identify off-target contigs, which are then removed or trimmed. The resulting plastid genome was then used as a reference for mapping the original reads back using Genieous Prime v.2023.2.1 (Biomatters Ltd., Auckland, New Zealand), allowing only for mapping of paired reads mapped nearby with a minimum overlap of 75 bp and a minimum overlap identity of 98%. The result was manually examined and corrected where necessary. Annotation was performed using GeSeq [23] and manually edited in Geneious Prime v.2023.2.1. Plastide sequences were aligned using MAFFT [24]. The alignment was checked and edited manually, if necessary, using Genieous Prime v.2023.2.1.

### 3.5. Assembly of GBS Data

GBS reads were clustered using the IPYRAD v.0.9.58 [18] pipeline with a clustering threshold of 0.85, according to [4]. In the initial output files generated by IPYRAD, a loci had to be present in 40 out of 210 samples. The resulting vcf file was filtered using vcftools v 0.1.16 [25], removing all indels and keeping only sites with a minor allele frequency of 0.02, a minimum depth of 10, and a maximum depth of 300. For phylogenetic analysis, SNPs were further filtered. To reduce the number of potentially variable but uninformative variants SNP, they needed to be present at least three times in the nine *C. bachofenii* individuals and must not be present in all species. Furthermore, the dataset only included loci and positions that were present in *C. bachofenii*. To locate positions in the VCF file that met the specified criteria, the R packages *adegenet* [26,27], *vcfR* [28], and *genomalicious* [29] were used. We then utilised vcftools to create a vcf file including only these positions and create a subset with only one-to-three individuals per population, excluding the *C.* cf. *heuffelianus* allotetraploids from Raca et al. [4]. This sample-filtered vcf file was then converted into phylip format using the python script vcf2phylip.py [30] and subjected to phylogenetic analysis.

### 3.6. Phylogenetic Inference

Bayesian phylogenetic inference (BI) was conducted in MrBayes 3.2.7 [31] for the chloroplasts. Two runs, each with-four chains were run for 1 million generations, specifying the respective model of sequence evolution and defining *C. malyi* as an outgroup. A tree was sampled every 100 generations. Converging log-likelihoods, potential scale-reduction factors for each parameter, and the inspection of tabulated model parameters in MrBayes suggested that stationary had been reached in all cases. The first 25% of trees of each run were discarded as burn-in.

For the genotyping-by-sequencing data published by Raca et al. [4], one-to-three individuals per population were selected. A newly collected population of *C. tommasinianus* from Albania was added, along with three samples of *C. siculus*. Maximum likelihood trees were estimated by IQ-tree v2.2.6 [32,33], applying the TVM + F + G4 model with 1000 bootstrap replications [34]. Modelfinder [35] was used to find the best-fit model for phylogenetic inference by determining the Bayesian information criterion. The resulting trees were used to infer a supernetwork in SplitsTree v4.18.2 [36].

### 3.7. Origin of Crocus bachofenii

Shared and species-specific alleles (private alleles) were inferred in R using the packages adegenet [26,27], vcfR [28], data.table [37], dplyr [38], tidyr [39], and tibble [40]. The vcf file, including all 210 individuals, was read in and converted into a genpop object. The latter one is a table including the position ID, SNP, and the count for the respective SNP per species. First, private alleles were determined for all species, as well as for the five different *C. vernus* and *C. heuffelianus* hybrids: *C.* cf. *heuffelianus* 2*n* = 18 SCC (Southern Carpathian clade), *C.* cf. *heuffelianus* 2*n* = 18 WCC (Western Carpathian clade), *C.* cf. *heuffelianus* 2*n* = 18 PIC (Pannonian–Illyric clade), *C.* cf. *heuffelianus* 2*n* = 20, and *C.* cf. *heuffelianus* 2*n* = 22. To focus on the composition of *C. bachofenii*, we then excluded all hybrids and the allotetraploid *C. neglectus* and kept only the diploid species, in addition to *C. bachofenii*. For this subset of private alleles, alleles shared by *C. bachofenii* with any of the other species, along with alleles exclusively shared by *C. bachofenii* with any of the other species (i.e., only present in two species), were counted. The same was done for the *C. bachofenii* and the three main clades consisting of *C. vernus*, *C. siculus,* and *C. neapolitanus* (cladeI), *C. bertiscensis*, *C. heuffelianus*, *C. kosaninii,* and *C. tommasinianus* (cladeII), and *C. etruscus* and *C. ilvensis* (cladeIII).

### 3.8. Inference of Clonal Reproduction

The shared heterozygosity (SH) index was calculated to infer the degree of clonal reproduction within *C. bachofenii* as described by [41]. It is based on the proportion of shared identically heterozygous SNPs of a pair of samples. The SH index was inferred for all samples within one species or allopolyploid lineage and plotted with ggplot2 [42] in R.

### 3.9. Cytological Analysis

Ten *C. bachofenii* individuals from Linos, Albania were cultivated in pots at IPK Gatersleben, and root tips were harvested in autumn. Metaphase spreads for FISH analysis were prepared following the methods described in Waminal et al. [43]. We skimmed the genome of *C. bachofenii* and nine other species in the *C*. ser. *Verni* for novel satellite repeats that can be used as cytogenetic markers. We used <1× whole-genome sequence short reads as input to the RepeatExplorer pipeline [44]. We developed oligonucleotide FISH probes for these three repeats by conjugating Cy3 (CroSat137), Cy5 (CroSat042), and FAM/FITC (CroSat144), respectively, at both the 5′ and 3′ ends of the oligonucleotides. In addition, we used the vertebrate-type (TTAGGG) telomeric repeat, which showed abundance in the genus *Crocus* [12], and the universal FISH probes for 45S rDNA and 5SrDNA (Table 3). The FISH was performed using a rapid method described by Waminal et al. [45].

### 3.10. Morphological Analysis

The morphological analysis was performed on 50 individuals per population, including the new species *C. bachofenii* (eight populations) and its morphologically similar relatives: *C. vernus* (three populations), *C. bertiscensis* (three populations), and *C. tommasinianus* (five populations). The data on *C. neapolitanus* [46] and *C. neglectus* morphology [5] were taken from the relevant literature source. Detailed information about the populations included in the morphological analysis is provided in Table S2. Quantitative analysis was derived from the measurements of 10 characters (corm width, fibre width, leaf width, perigone tube length, perigone segment length and width, stigma/anther ratio, anther length, stigma lobe, and sublobe length). Three meristic features were analysed as well (number of leaves, number of cataphylls, and number of flowers), while five qualitative characters were checked for their constancy (perigone tube color, outer perigone segment color, position of the stigma, anther color, and stigma color). Images and measurements were taken by a stereomicroscope Leica M205C, Wetzlar, Germany, equipped with Flexacam C3, Wetzlar, Germany and Leica Application Suite X software, Wetzlar, Germany.

### 3.11. Anatomical Analysis

The slides of the leaf cross-sections of *C. bachofenii* were made on a manual microtome [47]. The sections of 10 individuals from the type population were double-stained with safranin (1g of dye diluted in 100 mL of 50% ethanol) and alcian blue (1g of dye dissolved in 100 mL of distilled water, with a couple of phenolic crystals and three drops of glacial acetic acid). Stained sections were then dehydrated (series of 50%–70%–96%–100% alcohols), examined, and photographed on a Leica DM 1000 (Leica Microsystem, Wetzlar, Germany) microscope [10,48,49,50]. A total of 20 anatomical features were measured in ImageJ software, Madison, WI, USA [51]; the precise values are expressed in μm and μm^2^. The characters were related to the leaf blade (section height and width, arm length, white stripe width, section width/white stripe width ratio, and central parenchyma area), epidermal (adaxial epidermal cell height and width; abaxial epidermal cell height and width), mesophyll (palisade cell height and width, palisade tissue height; spongy cell height and width, spongy tissue height), and vascular tissue (number of vascular bundles, xylem, phloem, and sclerenchyma cap area surface).

## 4. Discussion

Since its taxonomic status was unclear at that point, we previously [4] referred to *C. bachofenii* as *C.* cf. *vernus*. The findings from this study indicated that *C. bachofenii* is a tetraploid involving *C. vernus* from the eastern distribution range in its origin. Phylogenetic trees based on GBS consistently grouped *C. bachofenii* as sister to *C. vernus*, *C. neapolitanus*, and *C. siculus*. Various methods, including GBS and phylogenetic markers, as reported by [4], did not indicate contributions from any lineage outside the *C. vernus* clade. The only indication for a contribution outside the *C. vernus* clade is the decreasing support value for the clade comprising *C. bertiscensis*, *C. heuffelianus*, *C. kosaninii*, and *C. tommasinianus* as soon as *C. bachofenii* is included. This led [4] to the hypothesis that *C. bachofenii* is likely an allotetraploid of *C. vernus* and an extinct parent, closely related to *C. vernus* and its two closest allies *C. siculus* and *C. neapolitanus*. Our investigations involving complete chloroplast sequences (instead of just a little part of it) confirmed the maternal contribution from eastern *C. vernus*.

Following a very conservative approach, we then checked with which species precisely *C. bachofenii* is sharing alleles. It is evident that the highest proportion of shared alleles is found in the *C. vernus* clade, including *C. siculus* and *C. neapolitanus* (Clade I), as well as in the clade comprising *C. heuffelianus*, *C. tommasinianus*, *C. bertiscensis*, and *C. kosaninii* (Clade II) (Table 1).

Propagation of *C. bachofenii* is mainly sexual, as indicated by the shared heterozygosity, which falls below the threshold of 0.9 (Figure 3). Shared heterozygosity above 0.9 typically characterises clonally propagated individuals [41]. The predominant sexual propagation of the tetraploid *C. bachofenii* indicates genetic divergence between parental subgenomes. This subgenome difference implies stable meiosis indicative of allopolyploidy. For autopolyploids, we would expect lower heterozygosity, indicating subgenome homogeneity and more problematic chromosome pairing. Moreover, the disomic pairing of chromosomes observed in the *C. bachofenii* karyotype also supports its allopolyploid origin. Nevertheless, definitive cytogenomic evidence of allopolyploidy could be obtained through genomic in situ hybridization using parental genomes as probes [52], or using comparative repeat profiles from chromosome-level whole-genome assemblies [53]. Numerous hemizygous repeat loci indicate chromosomal rearrangements, which may have existed in the parental genomes, since *C. vernus* exhibits heteromorphic karyotypes across different populations [12,54] or may be novel following allopolyploidization of *C. bachofenii*.

The new species, *C. bachofenii*, shares the same chromosome number (2*n* = 16; [4]) with *C. tommasinianus* and *C. neglectus* (2*n* = 16; [54]), differing from *C. bertiscensis* and *C. vernus* (2*n* = 12, [10] and 2*n* = 8 [54], respectively).

The distribution range, habitats, and/or elevation of *C. bachofenii* differ from all other *C*. ser. *Verni* species. *Crocus bachofenii* thrives in the clearings of beech forests, rocky pastures, or meadows, while *C. bertiscensis* inhabits the alpine pastures and meadows above 1700 m a.s.l., in siliceous substrate [10,55,56]. *Crocus vernus* can be found in calcareous pastures within the beech belt, as well as *C. bachofenii*. However, its distribution range is shifted to the north of the Albanian Alps (1200–1700 m a.s.l.), sharing its habitat altitudinally with *C. bertiscensis* in this region [9,10,56]. Geographically, the closest locality of *C. bachofeni* is about 65 km away from the closest locality of *C. vernus* and 73 km away from the closest locality of *C. bertiscensis*. Of the species of *C*. ser. *Verni*, only *C. tommasinianus* is located ca. 3.5 km away from the closest locality of *C. bachofenii*. However, *C. bachofenii* is growing above (1300–1700 m a.s.l.) the distribution range of *C. tommasinianus* (300–1000 m a.s.l.). An occurrence at a higher elevation [11] was reported for *C. tommasinianus* in Albania based on a specimen (nr. 4715) collected from Kunora e Lurës on 2 August 1959. However, the species’ typical habitat at lower elevations casts doubt on its occurrence in the high-altitude of Lura Mt. Specimens collected later on 15 April 2013, by L. Shuka and R. Bachofen, and again on 13 April 2024, by D. Shuka in this area underwent detailed morpho-anatomical and genetic examinations. These studies identified the specimens as *C. bachofenii*, contradicting Meyer’s [11] report. Consequently, the localities in Figure S5 represent the first confirmed record of *C. tommasinianus* occurrence in Albania.

## Figures and Tables

**Table 1:** Alleles shared with *C. bachofenii*.

Species	No. of Shared Alleles		No. of Exclusively Shared Alleles	
*C. vernus* (32)	25,851	(84.77%)	1777	(5.83%)
*C. heuffelianus* (18)	21,187	(69.47%)	550	(1.80%)
*C. tommasinianus* (29)	18,412	(60.38%)	289	(0.95%)
*C. bertiscensis* (7)	15,078	(49.44%)	155	(0.51%)
*C. neapolitanus* (2)	13,391	(43.91%)	66	(0.22%)
*C. siculus* (4)	11,193	(36.70%)	39	(0.13%)
*C. kosaninii* (3)	10,328	(33.87%)	85	(0.28%)
*C. etruscus* (2)	9247	(30.32%)	18	(0.06%)
*C. ilvensis* (2)	8695	(28.51%)	4	(0.01%)
*C. longiflorus* (1)	3829	(12.56%)	47	(0.15%)
**Clades**	**No. of shared alleles**		**No. of exclusively shared alleles**	
CladeI: *C. vernus*—*C. neapolitanus*—*C. siculus* (38)	26,483	(86.84%)	3276	(10.74%)
CladeII: *C. heuffelianus*—*C. bertiscensis*—*C. tommasinianus*—*C. kosaninii* (57)	26,671	(87.46%)	2690	(8.82%)
CladeIII: *C. etruscus*—*C. ilvensis* (4)	10,909	(35.77%)	70	(0.23%)
CladeI + CladeII	30,378	(99.61%)	17,482	(57.33%)

Number of samples is provided in parenthesis.

**Table 2:** Morphological comparison of *C. bachofenii* with *C. vernus*, *C. bertiscensis*, *C. tommasinianus*, *C. neapolitanus*, and *C. neglectus*.

	*C. bachofenii*	*C. vernus*	*C. bertiscensis*	*C. tommasinianus*	*C. neapolitanus*	*C. neglectus*
Corm width (mm)	10.60 ± 2.77	12.80 ± 2.70	6.80 ± 0.80	10.40 ± 1.70	11–13	13.9 ± 1.9
Fibre width (mm)	0.10 ± 0.04	0.08 ± 0.03	0.08 ± 0.03	0.11 ± 0.02	0.06–0.02	0.10 ± 0.02
Number of leaves	2–5, usually 3	2–4, usually 3	1–3, usually 2	2–5, usually 3	2–4, usually 3	–
Leaf width (mm)	2.93 ± 0.30	1.83 ± 0.50	3.20 ± 0.50	1.76 ± 0.90	(2–) 4–8	–
Number of cataphylls	3–5, usually 4	3–4, usually 3	2–4, usually 3	3–4, usually 3	2–3	–
Perigone tube length (mm)	44.30 ± 7.50	43.60 ± 10.60	33.10 ± 5.90	62.30 ± 10.60)	40–100	60.7 ± 21.9
Perigone tube color	Deep violet-to-violet	Lilac or white	Lilac or deep purple	White or whitish	Lilac in upper part	Concolor
Number of flowers	1, rarely 2	1, rarely 2	1	1, rarely 2	1 (–2)	1 (–2)
Perigone segment length (mm)	38.50 ± 1.00	21.90 ± 0.50	27.22 ± 3.14	26.7 ± 2.00	25–55	34.8 ± 4.7
Perigone segment width (mm)	14.50 ± 1.50	6.40 ± 1.00	8.88 ± 1.25	11.27 ± 1.50	9–20	–
Outer perigone segment color	Lilac-to-deep lilac at the base and lilac-to-violet above	White, white with lilac stripes, or completely lilac	Lilac at the base, with a dark violet heart- or V-shaped patch at the apex of perigone segments	Pale lilac-to-lilac	Purple-to-deep purple	Lilac, rarely white
Position of the stigma	Longer than anthers (rarely equal to)	Shorter than anthers	Equal to or longer than anthers (rarely shorter)	Equal to or longer than anthers (rarely shorter)	Equal to or longer than anthers	Longer than anthers (rarely equal to)
Stigma/anther ratio (mm)	1.47 ± 2.50	−8.40 ± 3.90	1.11 ± 3.80	1.20 ± 2.30	1.10 ± 3.90	3.7 ± 2.1
Anther length (mm)	15.67 ± 3.00	9.30 ± 1.60	8.13 ± 1.11	10.40 ± 1.50	11.80 ± 0.29	12.6 ± 1.7
Anther color (mm)	Yellow-to-orange	Yellow	Yellow	Yellow	Yellow	Yellow
Stigma lobes length (mm)	2.34 ± 0.53	1.38 ± 0.75	1.62 ± 0.55	1.00 ± 0.09	–	–
Stigma sublobes length (mm)	Incised for 0.24 ± 0.01	Incised for 0.23 ± 0.22	Incised for 0.31 ± 0.30	Incised for 0.90 ± 0.32	Incised for 0.06 ± 0.02	Incised for 0.13 ± 0.08
Stigma color	Orange (rarely yellow)	Orange	Orange	Orange	Orange	Orange

Measurements are expressed through the Mean ± StDev, or Min–Max values.

**Table 3:** List of oligonucleotide FISH probes used in this study.

Name	Length (bp)	Sequence	5′ Modification	3′ Modification	Reference
CroSat137a	30	RAGCTGCTTAGAGTGCTATAGRAATGCAAC	Cy3	Cy3	This study
Crosat137b	30	GCTTSGATCAAACTGGGCYAAGATGCTCCC	Cy3	Cy3	This study
CroSat042a	30	CATGACTTGTAAAAACRGAGTYTTGCAAGC	Cy5	Cy5	This study
CroSat042b	30	GAAGATATCTACTCAAATCACACTAAAATA	Cy5	Cy5	This study
CroSat144a	30	TCTCCCGGAAACTCGTTTCGAGGCCTACCT	FAM	FITC	This study
CroSat144b	30	CATCGCGGACGGTTTCGGTCGTTACGTTGA	FAM	FITC	This study
TTAGGG	29	TTAGGGTTAGGGTTAGGGTTAGGGTTAGG	Cy3	Cy3	This study
18SrDNA_UniOP_1	28	CCGGAGAGGGAGCCTGAGAAACGGCTAC	Cy5	Cy5	Waminal et al. (2018) [45]
18SrDNA_UniOP_2	26	ATCCAAGGAAGGCAGCAGGCGCGCAA	Cy5	Cy5	Waminal et al. (2018) [45]
18SrDNA_UniOP_3	28	GGGCAAGTCTGGTGCCAGCAGCCGCGGT	Cy5	Cy5	Waminal et al. (2018) [45]
18SrDNA_UniOP_4	27	TCGAAGACGATYAGATACCGTCSTAGT	Cy5	Cy5	Waminal et al. (2018) [45]
5SrDNA_UniOP_1	30	GGGTGCGATCATACCAGCACTAGAGCACCG	FAM	FITC	Waminal et al. (2018) [45]
5SrDNA_UniOP_2	29	CCCATCAGAACTCCGAAGTTAAGCGTGCT	FAM	FITC	Waminal et al. (2018) [45]
5SrDNA_UniOP_3	24	GCGAGAGTAGTACTAGGATGGGTG	FAM	FITC	Waminal et al. (2018) [45]
5SrDNA_UniOP_4	26	CCTGGGAAGTMCTCGTGTTGCAYYCC	FAM	FITC	Waminal et al. (2018) [45]

## Data Availability

All data are available within the article and its Supplementary Materials.

## Supplementary Materials

The following supporting information can be downloaded at: https://www.mdpi.com/article/10.3390/plants14050741/s1, Figure S1: Comparative repeat quantification in *C.* ser. *Verni* as generated by RepeatExplorer. Satellite repeats that were used as FISH probes are noted; Figure S2: Distribution of six DNA repeats in *C. bachofenii* root metaphase chromosomes. This image was used to generate the karyogram presented in Figure 4. (A) 45S rDNA, (B) 5S rDNA, (C) vertebrate-type (TTAGGG) telomeric repeat, (D) CroSat137, (E) CroSat042, and (F) CroSat144. Bar = 10 µm; Figure S3: The corms and tunic details of *C. bachofenii*: fibres connected with thin (semitransparent) (a,b) and thicker membranes (c), *C. tommasinianus* (d), and *C. vernus* (e); Figure S4: The comparative herba collage of *C. bachofenii* (a) and its relatives: *C. neapolitanus* (b), *C. vernus* (c), *C. bertiscensis* (d), and *C. tommasinianus* (e); Figure S5: The distribution map of the species of *C.* ser. *Verni* in Albania; Table S1: Private alleles; Table S2: Information on the populations of various species in *C.* ser. *Verni* included in the morphological analysis; Table S3: Information about the individuals of *C.* ser. *Verni* that underwent genotyping-by-sequencing (GBS).

## References

[1] Doyle J.J., Sherman-Broyles S. (2017). Double trouble: Taxonomy and definitions of polyploidy. New Phytol..

[2] Żabicka J., Kirschey T., Migdałek G., Słomka A., Kuta E. (2023). Genetic variation versus morphological variability in European peatland violets (*Viola epipsila*—V. *palustris* group). Biology.

[3] Edger P.P., McKain M.R., Bird K.A., Vanburen R. (2018). Subgenome assignment in allopolyploids: Challenges and future directions. Curr. Opin. Plant Biol..

[4] Raca I., Blattner F.R., Waminal N.E., Kerndorff H., Ranđelović V., Harpke D. (2023). Disentangling *Crocus* series *Verni* and its polyploids. Biology.

[5] Harpke D., Carta A., Tomović G., Ranđelović V., Ranđelović N., Blattner F.R., Peruzzi L. (2015). Phylogeny, karyotype evolution and taxonomy of *Crocus* series *Verni* (Iridaceae). Plant Syst. Evol..

[6] Elshire R.J., Glaubitz J.C., Sun Q., Poland J.A., Kawamoto K., Buckler E.S., Mitchell S.E. (2011). A robust, simple genotyping-by-sequencing (GBS) approach for high diversity species. PLoS ONE.

[7] Le Comber S.C., Ainouche M.L., Kovarik A., Leitch A.R. (2010). Making a functional diploid: From polysomic to disomic inheritance. New Phytol..

[8] Lv Z., Addo Nyarko C., Ramtekey V., Behn H., Mason A.S. (2024). Defining autopolyploidy: Cytology, genetics, and taxonomy. Am. J. Bot..

[9] Shuka L., Zekaj Z., Mullaj A. Biogeographical records of species of the genus *Crocus* L. in Albania. Proceedings of the 5th Balkan Botanical Congress.

[10] Raca I., Harpke D., Shuka L., Ranđelović V. (2022). A new species of *Crocus* ser. *Verni* (Iridaceae) with 2*n* = 12 chromosomes from the Balkans. Plant Biosyst.-Int. J. Deal. All Asp. Plant Biol..

[11] Meyer F.K. (2011). Beitrage zur Flora von Albanien.

[12] Waminal N.E., Blattner F., Harpke D. (2024). The *Crocus* panrepeatome reveals the links between whole-genome duplications, repeat bursts, and descending dysploidy. Res. Sq..

[13] Buzo K. (1991). Bimësia e Kullotave dhe e Livadheve Natyrore të Shqiperisë.

[14] Shuka L., Çullaj A., Shumka S., Miho A., Duka S., Bachofen R. (2011). The spatial and temporal variability of limnological properties of Bovilla Reservoir (Albania). Water Resour. Manag..

[15] Meyer M., Kircher M. (2010). Illumina sequencing library preparation for highly multiplexed target capture and sequencing. Cold Spring Harb. Protoc..

[16] Andrews S. (2010). FastQC: A Quality Control Tool for High Throughput Sequence Data. http://www.bioinformatics.babraham.ac.uk/projects/fastqc/.

[17] Martin M. (2011). Cutadapt removes adapter sequences from high-throughput sequencing reads. EMBnet.J..

[18] Eaton D.A.R., Overcast I. (2020). ipyrad: Interactive assembly and analysis of RADseq datasets. Bioinformatics.

[19] Jin J.-J., Yu W.-B., Yang J.-B., Song Y., Depamphilis C.W., Yi T.-S., Li D.-Z. (2020). GetOrganelle: A fast and versatile toolkit for accurate de novo assembly of organelle genomes. Genome Biol..

[20] Langmead B., Salzberg S.L. (2012). Fast gapped-read alignment with Bowtie 2. Nat. Methods.

[21] Bankevich A., Nurk S., Antipov D., Gurevich A.A., Dvorkin M., Kulikov A.S., Lesin V.M., Nikolenko S.I., Pham S., Prjibelski A.D. (2012). SPAdes: A new genome assembly algorithm and its applications to single-cell sequencing. J. Comput. Biol..

[22] Camacho C., Coulouris G., Avagyan V., Ma N., Papadopoulos J., Bealer K., Madden T.L. (2009). BLAST+: Architecture and applications. BMC Bioinform..

[23] Tillich M., Lehwark P., Pellizzer T., Ulbricht-Jones E.S., Fischer A., Bock R., Greiner S. (2017). GeSeq—Versatile and accurate annotation of organelle genomes. Nucleic Acids Res..

[24] Katoh K., Standley D.M. (2013). MAFFT Multiple Sequence Alignment Software Version 7: Improvements in performance and usability. Mol. Biol. Evol..

[25] Danecek P., Auton A., Abecasis G., Albers C.A., Banks E., Depristo M.A., Handsaker R.E., Lunter G., Marth G.T., Sherry S.T. (2011). The variant call format and VCFtools. Bioinformatics.

[26] Jombart T., Ahmed I. (2011). *adegenet 1.3-1*: New tools for the analysis of genome-wide SNP data. Bioinformatics.

[27] Jombart T. (2008). *adegenet*: A R package for the multivariate analysis of genetic markers. Bioinformatics.

[28] Knaus B.J., Grünwald N.J. (2017). VCFR: A package to manipulate and visualize variant call format data in R. Mol. Ecol. Resour..

[29] Thia J.A. (2019). *genomalicious*: Serving up a smorgasbord of R functions for performing and teaching population genomic analyses. BioRxiv.

[30] Ortiz E.M. (2019). vcf2phylip v2.0: Convert a VCF Matrix into Several Matrix Formats for Phylogenetic Analysis. https://zenodo.org/records/2540861.

[31] Ronquist F., Teslenko M., van der Mark P., Ayres D.L., Darling A., Höhna S., Larget B., Liu L., Suchard M.A., Huelsenbeck J.P. (2012). MrBayes 3.2: Efficient bayesian phylogenetic inference and model choice across a large model space. Syst. Biol..

[32] Nguyen L.-T., Schmidt H.A., von Haeseler A., Minh B.Q. (2014). IQ-TREE: A fast and effective stochastic algorithm for estimating maximum-likelihood phylogenies. Mol. Biol. Evol..

[33] Minh B.Q., Schmidt H.A., Chernomor O., Schrempf D., Woodhams M.D., von Haeseler A., Lanfear R. (2020). IQ-TREE 2: New models and efficient methods for phylogenetic inference in the genomic era. Mol. Biol. Evol..

[34] Hoang D.T., Chernomor O., von Haeseler A., Minh B.Q., Vinh L.S. (2017). UFBoot2: Improving the ultrafast bootstrap approximation. Mol. Biol. Evol..

[35] Kalyaanamoorthy S., Minh B.Q., Wong T.K.F., von Haeseler A., Jermiin L.S. (2017). ModelFinder: Fast model selection for accurate phylogenetic estimates. Nat. Methods.

[36] Huson D.H., Bryant D. (2006). Application of phylogenetic networks in evolutionary studies. Mol. Biol. Evol..

[37] Barrett T., Dowle M., Srinivasan A., Gorecki J., Chirico M., Hocking T., Schwendinger B., Krylov I. (2024). data.table: Extension of ‘data.frame’, R Package Version 1.15.99. https://Rdatatable.gitlab.io/data.table.

[38] Wickham H., François R., Henry L., Müller K., Vaughan D. (2023). dplyr: A Grammar of Data Manipulation, R Package Version 1.1.4. https://github.com/tidyverse/dplyr.

[39] Wickham H., Vaughan D., Girlich M. (2024). tidyr: Tidy Messy Data, R Package Version 1.3.1. https://tidyr.tidyverse.org.

[40] Müller K., Wickham H. (2023). tibble: Simple Data Frames. https://tibble.tidyverse.org/.

[41] Yu L., Stachowicz J.J., Dubois K., Reusch T.B.H. (2023). Detecting clonemate pairs in multicellular diploid clonal species based on a shared heterozygosity index. Mol. Ecol. Resour..

[42] Wickham H. (2016). ggplot2: Elegant Graphics for Data Analysis.

[43] Waminal N.E., Pellerin R.J., Kang S.-H., Kim H.H. (2021). Chromosomal mapping of tandem repeats revealed massive chromosomal rearrangements and insights into *Senna tora* dysploidy. Front. Plant Sci..

[44] Novák P., Neumann P., Macas J. (2020). Global analysis of repetitive DNA from unassembled sequence reads using RepeatExplorer2. Nat. Protoc..

[45] Waminal N.E., Pellerin R.J., Kim N.-S., Jayakodi M., Park J.Y., Yang T.-J., Kim H.H. (2018). Rapid and efficient FISH using pre-labeled oligomer probes. Sci. Rep..

[46] Milović M. (2016). Rod *Crocus* L. (Iridaceae) u flori Hrvatske. J. Croat. Bot. Soc..

[47] Gligorijević S., Pejčinović D. (1983). Contribution to the methodology of anatomical sections preparation. Acta Biol. Et Med. Exp..

[48] Raca I., Ljubisavljević I., Jušković M., Ranđelović N., Ranđelović V. (2017). Comparative anatomical study of the taxa from series *Verni* Mathew (*Crocus* L.) in Serbia. Biol. Nyssana.

[49] Raca I., Jovanovic M., Ljubisavljevic I., Juskovic M., Randelovic V. (2019). Morphological and leaf anatomical variability of *Crocus* cf. *heuffelianus* Herb.(Iridaceae) populations from the different habitats of the Balkan Peninsula. Turk. J. Bot..

[50] Raca I. (2021). Taxonomy and Phylogeny of Series Verni Mathew (Crocus L.) in Southeastern Europe—Morpho-Anatomical, Cytological and Molecular Approach. Ph.D. Thesis.

[51] Schneider C.A., Rasband W.S., Eliceiri K.W. (2012). NIH Image to ImageJ: 25 years of image analysis. Nat. Methods.

[52] Keller E.R.J., Schubert I., Fuchs J., Meister A.J.T., Genetics A. (1996). Interspecific crosses of onion with distant *Allium* species and characterization of the presumed hybrids by means of flow cytometry, karyotype analysis and genomic in situ hybridization. Theor. Appl. Genet..

[53] Session A.M., Rokhsar D.S. (2023). Transposon signatures of allopolyploid genome evolution. Nat. Commun..

[54] Brighton C.A. (1976). Cytological problems in the genus *Crocus* (Iridaceae): I. *Crocus vernus* aggregate. Kew Bull..

[55] Shuka D., Tan K., Hallaçi B., Shuka L. (2020). Additions to the flora of North Albania. Phytol. Balc..

[56] Shumka S., Shumka L., Špoljar M., Shuka L. (2024). Evidence of climate change and the conservation needed to halt the further deterioration of small glacial lakes. Climate.

