# Supplementary figures and images for: An Integrative Systematic Approach Reveals a New Species of Crocus Series Verni (Iridaceae) Endemic to Albania
Source: Plants (Basel). 2025 Feb 28;14(5):741. doi: 10.3390/plants14050741 (PMC11901598; doi:10.3390/plants14050741)

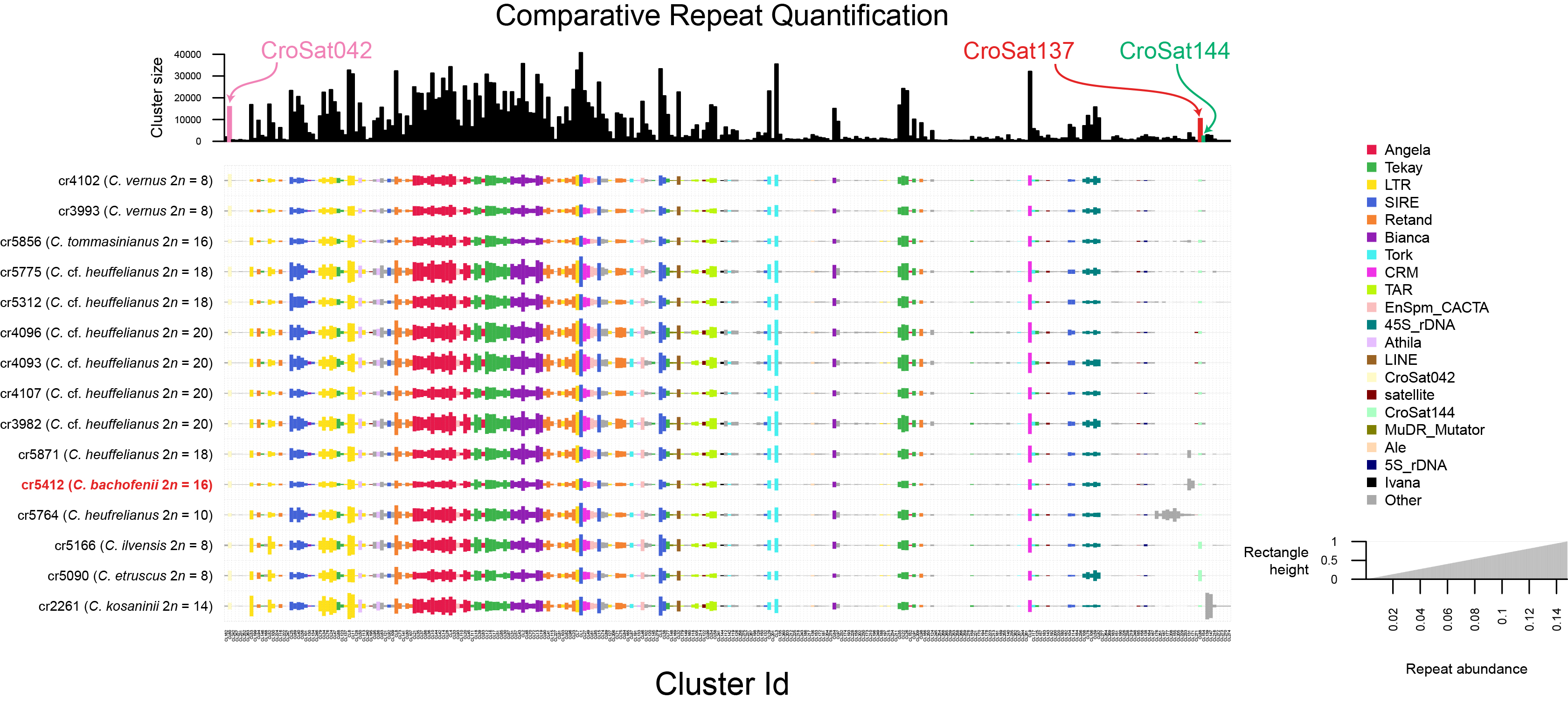

Supplement: Supplementary file 1 [file plants-14-00741-s001.zip › Figure_S1.jpg]

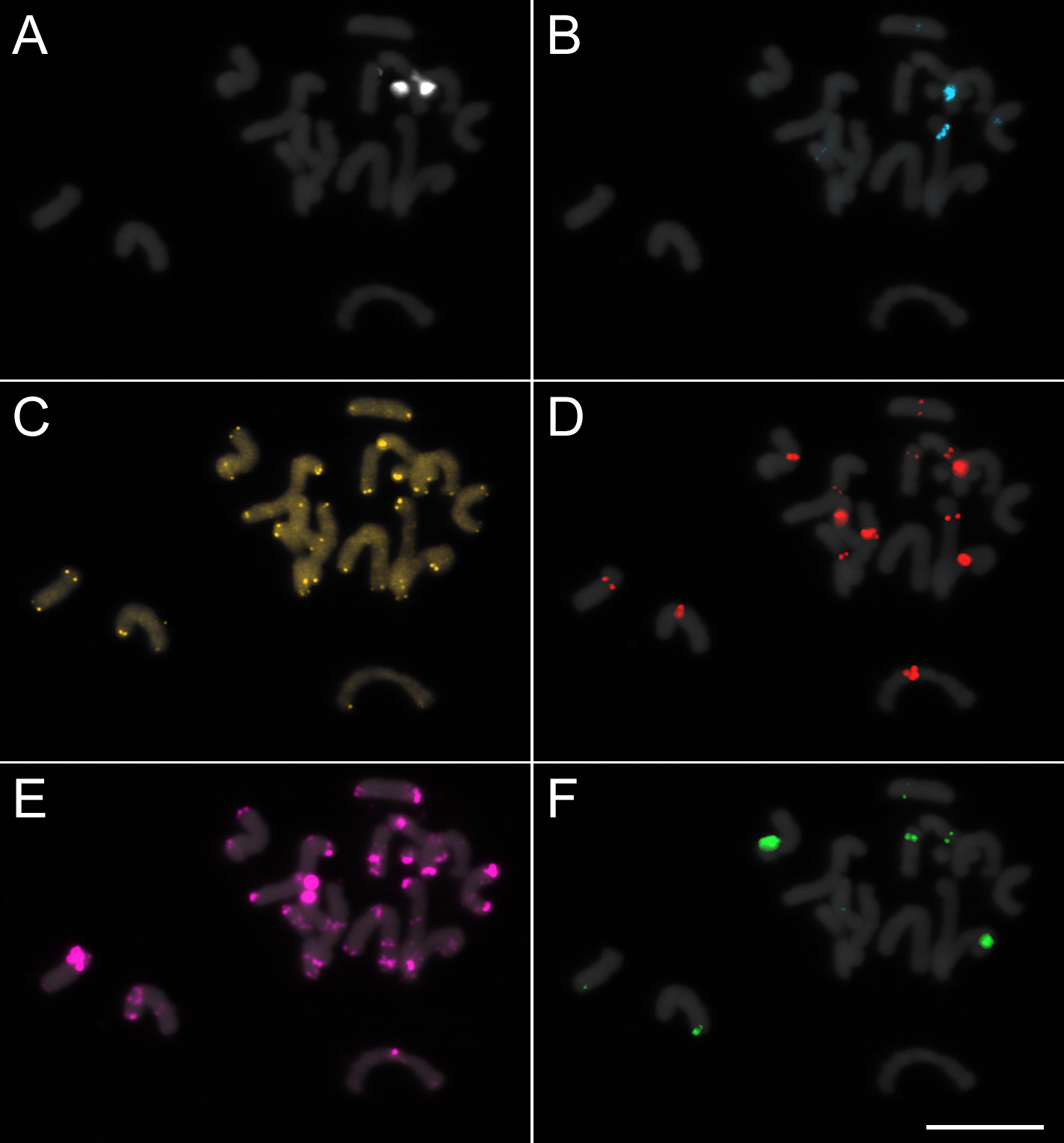

Supplement: Supplementary file 1 [file plants-14-00741-s001.zip › Figure_S2.tif]

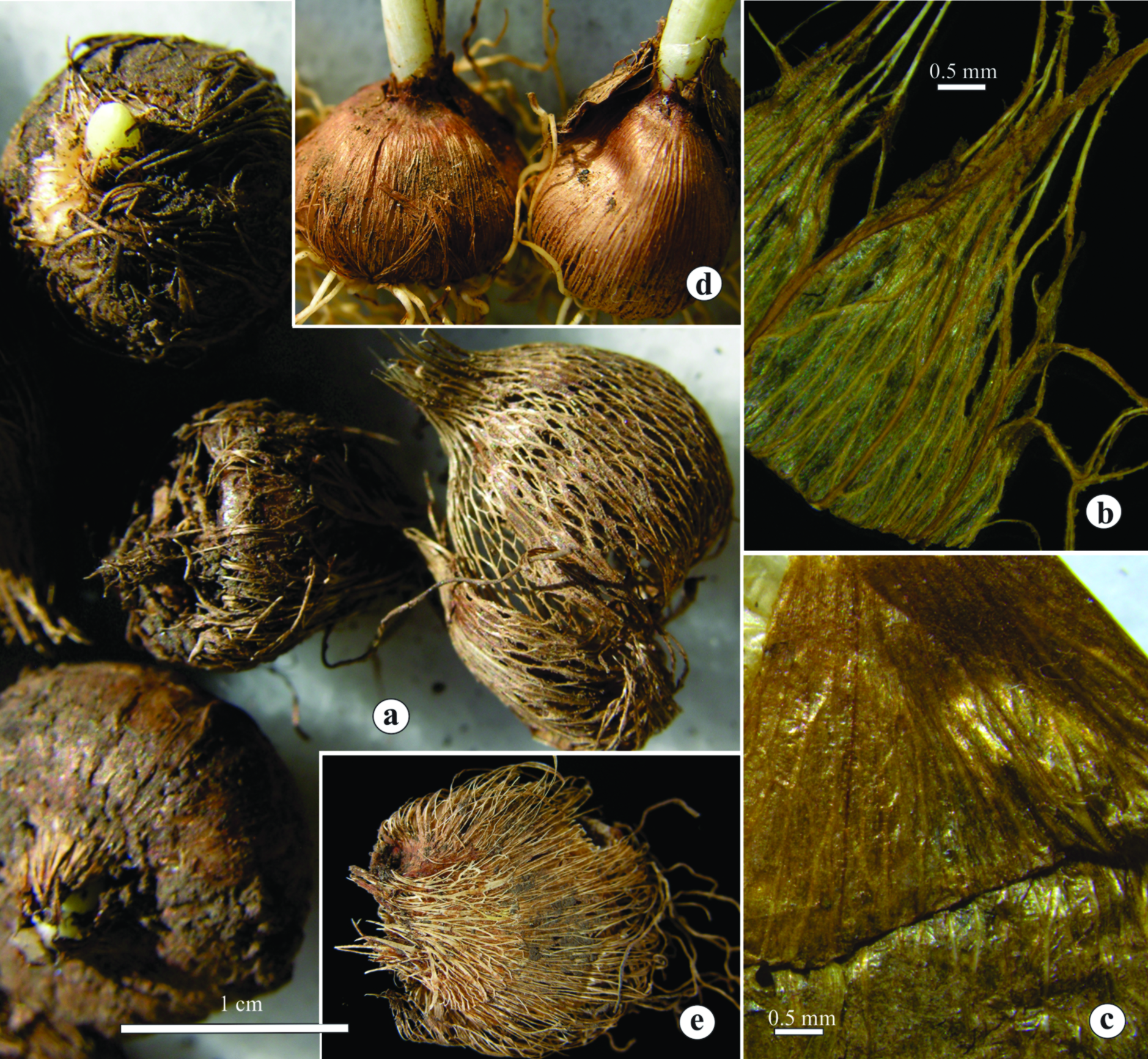

Supplement: Supplementary file 1 [file plants-14-00741-s001.zip › Figure_S3.jpg]

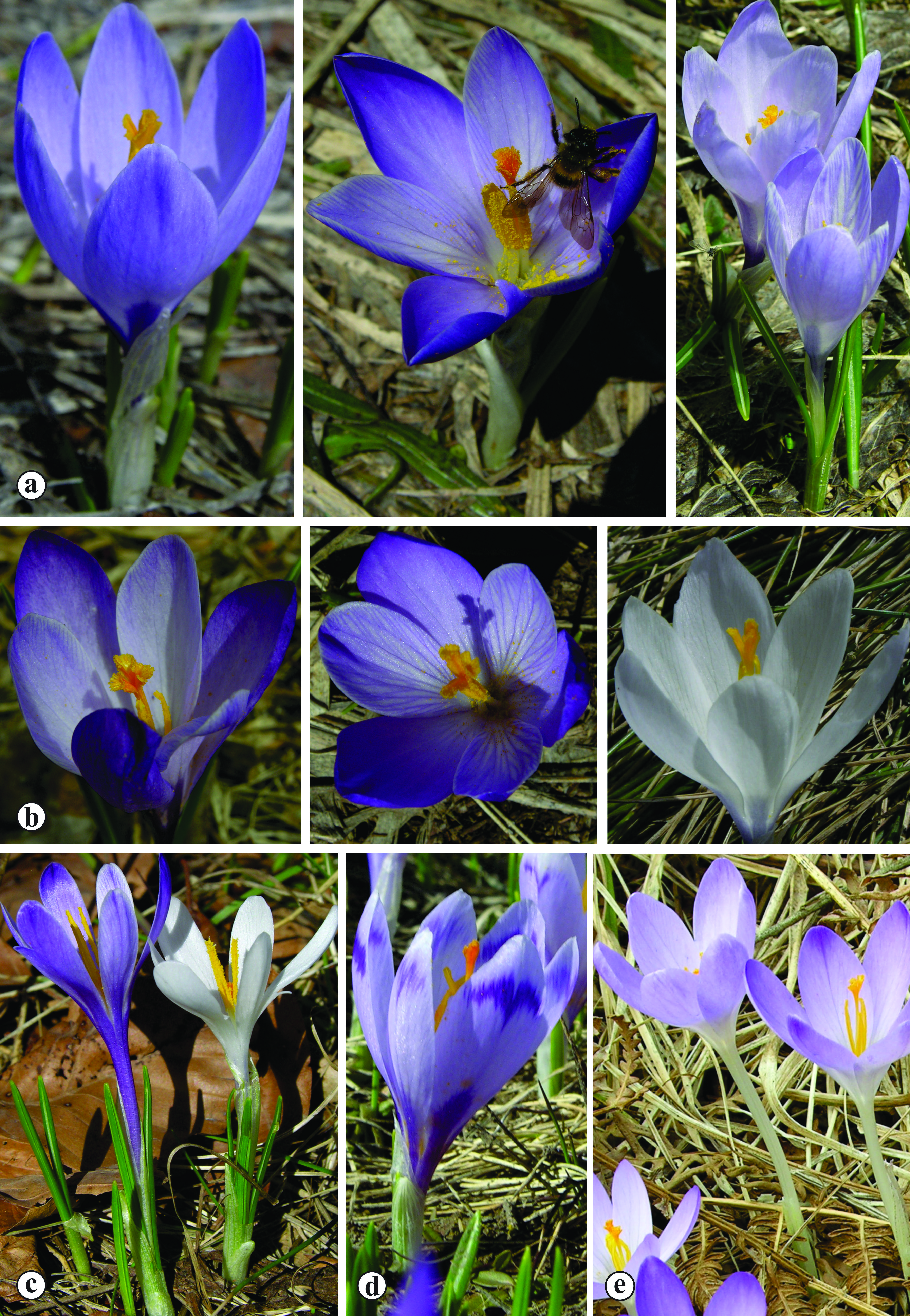

Supplement: Supplementary file 1 [file plants-14-00741-s001.zip › Figure_S4.jpg]

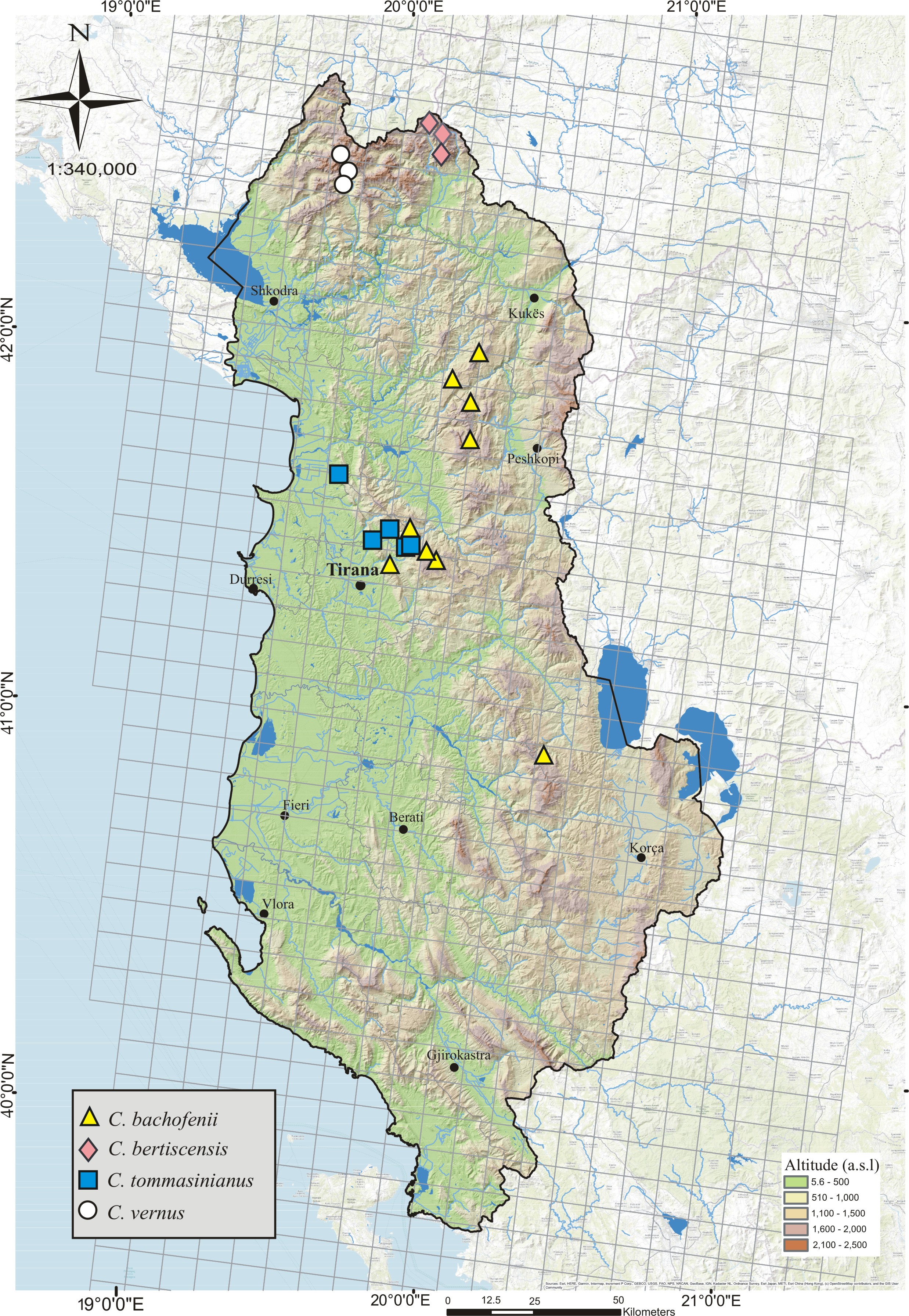

Supplement: Supplementary file 1 [file plants-14-00741-s001.zip › Figure_S5.jpg]
